# Cultural Engagement and Incidence of Cognitive Impairment: A 6-year Longitudinal Follow-up of the Japan Gerontological Evaluation Study (JAGES)

**DOI:** 10.2188/jea.JE20190337

**Published:** 2021-10-05

**Authors:** Akiho Sugita, Ling Ling, Taishi Tsuji, Katsunori Kondo, Ichiro Kawachi

**Affiliations:** 1 Faculty of Medicine, Chiba University, Chiba, Japan; 2 Graduate School of Medical and Pharmaceutical Sciences, Chiba University, Chiba, Japan; 3 Faculty of Health and Sport Sciences, University of Tsukuba, Tokyo, Japan; 4 Center for Preventive Medical Sciences, Chiba University, Chiba, Japan; 5 Center for Gerontology and Social Science, National Center for Geriatrics and Gerontology, Aichi, Japan; 6 Department of Social and Behavioral Sciences, Harvard T.H. Chan School of Public Health, Boston, Massachusetts, United States

**Keywords:** prevention, dementia, cultural activity, reading, hobby

## Abstract

**Background:**

Active engagement in intellectually enriching activities reportedly lowers the risk of cognitive decline; however, few studies have examined this association, including engagement in traditional cultural activities. This study aimed to elucidate the types of cultural engagement associated with lower risk of cognitive impairment.

**Methods:**

We examined the association between cultural engagement and cognitive impairment using Cox proportional hazards models in a cohort of 44,985 participants (20,772 males and 24,213 females) aged 65 years or older of the Japan Gerontological Evaluation Study from 2010 to 2016. Intellectual activities (eg, reading books, magazines, and/or newspapers), creative activities (eg, crafts and painting), and traditional cultural activities (eg, poetry composition [*haiku*], calligraphy, and tea ceremony/flower arrangement) were included among cultural engagement activities.

**Results:**

Over a follow-up period of 6 years, incident cognitive disability was observed in 4,198 respondents (9.3%). After adjusting for potential confounders, such as depression and social support, intellectual activities were protectively associated with the risk of cognitive impairment (hazard ratio [HR] for those who read and stated that reading was their hobby, 0.75; 95% confidence interval [CI] 0.66–0.85 and HR for those who read but did not consider reading a hobby, 0.72; 95% CI, 0.65–0.80). Engagement in creative activities was also significantly correlated with lower risk of cognitive impairment (crafts: HR 0.71; 95% CI, 0.62–0.81 and painting: HR 0.80; 95% CI, 0.66–0.96). The association between traditional cultural activities and the risk of cognitive impairment was not statistically significant.

**Conclusions:**

Engagement in intellectual and creative activities may be associated with reduced risk of dementia.

## INTRODUCTION

Societies experiencing rapid population aging are grappling with the parallel rise in cases of dementia. There has been a rapid growth in the number of people with dementia not only in high-income countries but also low- and middle-income countries.^1^

In Japan, the situation is even more pressing. The estimated number of people aged 65 or older stood at 35.89 million as of October 2019, accounting for 28.4% of the nation’s total population,^2^ with both figures hitting record highs. The share of the older population was the highest among 263 countries and regions in the world.^3^ According to a government report, in 2012, one in seven older adults aged 65 and above had dementia, with this figure estimated to reach one in five people in 2025.^4^

Current pharmacological treatment of dementia (eg, cholinesterase inhibitors and N-methyl D-aspartate receptor antagonists) is only able to address the relief of symptoms or prevent the progression of dementia; the damage to brain cells is permanent. Therefore, the only viable population approach to dementia is to focus on prevention. Specifically, modifiable factors, such as cognitive training, exercise, and strong social support may be associated with increased brain and cognitive reserve.^1^

In observational studies, engagement in mentally challenging activities has been suggested to be protectively associated with dementia risk.^5^^–^^12^ The effects of leisure activities (eg, reading, radio and TV, gardening, puzzles, social visits, going to the theater, cinema, and museums)^5^^–^^11^ and computer use^11^^,^^12^ have been evaluated.

However, such studies have not been without limitations. For example, these studies did not control for major confounders for cultural engagement, such as depression,^6^^–^^8^ or visual or hearing impairment,^6^^–^^9^^,^^11^ as well as receiving/giving social support.^5^^–^^12^ Additionally, some of the “intellectual activities” and/or “cultural activities” examined in previous studies included physical activities (eg, going to the theater, cinema, and museums),^5^^,^^6^ playing games, or simple tasks.^7^ Since existing studies have shown associations between such activities and dementia or cognitive impairment,^9^^,^^10^^,^^13^ we focused on the concept of cultural engagement in this study.

As for participation in uniquely Japanese forms of cultural engagement, including poetry composition (*haiku*), calligraphy, tea ceremony/flower arrangement, the influences on the risk of dementia remain unclear. Japan has achieved the world’s longest healthy life expectancy and life expectancy for both sexes in 2013.^14^ Engagement in traditional cultural activities is possibly one of the reasons why Japanese older people can maintain their health. In a cohort of 27 patients with neurocognitive disorders, an intervention based on a flower arrangement program improved dysfunctions in visuospatial memory and recognition in patients.^15^ However, no study has examined the long-term association between engagement in Japanese cultural activities and the risk of cognitive deterioration among Japanese older adults living almost independent lives at baseline.

In this study, we hypothesized that there is an association between cultural engagement (including uniquely Japanese forms of cultural activities) and cognitive impairment. We focused on three specific aspects: intellectual activities (eg, reading books, magazines, and/or newspapers), creative activities (eg, crafts and painting), and traditional cultural activities (eg, poetry composition [*haiku*], calligraphy, and tea ceremony/flower arrangement). We sought to investigate whether forms of cultural engagement are associated with lower risk of cognitive impairment, as these associations have not been fully identified yet.

## METHODS

### Study sample

The Japan Gerontological Evaluation Study (JAGES) is a nationwide cohort study established in 2010 to examine prospectively the predictors of healthy aging.^16^ A total of 95,827 community-dwelling people aged 65 or older in 13 municipalities within Japan were mailed our baseline questionnaire from August 2010 to January 2012. Of the 62,426 individuals who responded to the invitation (response rate 65.1%), 5,739 respondents were removed owing to missing/invalid information about gender and/or age. After excluding an additional 1,444 respondents who did not agree to the use of their data and those with invalid ID numbers, 55,243 participants remained available for analyses.

**Figure 1.**
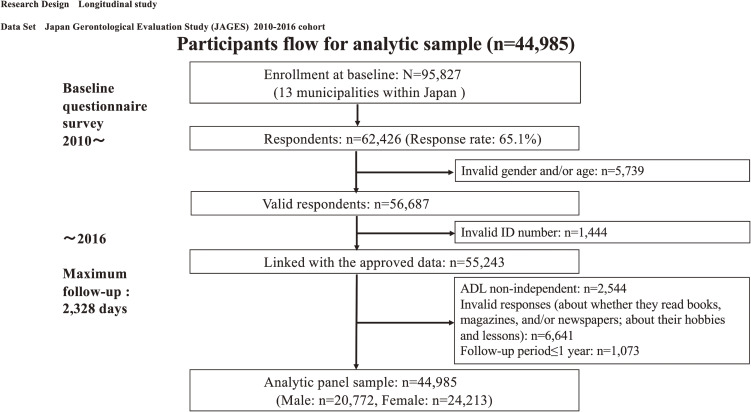
Participants flow for analytic sample.

The questionnaire survey inquired about respondents’ personal characteristics, health status, and health habits. As shown in Figure 1, of the 55,243 eligible participants from the baseline survey, we excluded 2,544 who were not independent in their activities of daily living (ADL). We also removed 6,641 respondents who failed to answer the section of the survey asking about their engagement in cultural activities.

In order to address the possibility of reverse causality (ie, low engagement in cultural activities being a marker for pre-existing cognitive decline), we performed analyses excluding a further 1,073 respondents whose follow-up periods were ≤1 year.

Finally, our longitudinal sample was *n* = 44,985 (male: *n* = 20,772, female: *n* = 24,213). Table 1 summarizes the characteristics of the final analytic sample. Males made up 46.2% of the sample; the mean age was 73.6 (standard deviation, 5.81) years. For females, the mean age was 73.9 (standard deviation, 5.97) years.

**Table 1.** Characteristics of respondents by gender (baseline survey in 2010)

Characteristics	All (*n* = 44,985)		Male (*n* = 20,772)		Female (*n* = 24,213)		*P*-value

	*n*	%	*n*	%	*n*	%	
**Level of cognitive impairment**							<0.01
0,1	40,787	90.7	18,951	91.2	21,836	90.2	
2–7	4,198	9.3	1,821	8.8	2,377	9.8	
Total	44,985	100.0	20,772	100.0	24,213	100.0	
**Age, years**							<0.01
65–69	12,928	28.7	6,112	29.4	6,816	28.2	
70–74	13,902	30.9	6,409	30.9	7,493	30.9	
75–79	10,171	22.6	4,671	22.5	5,500	22.7	
79–84	5,541	12.3	2,575	12.4	2,966	12.2	
≥85	2,443	5.4	1,005	4.8	1,438	5.9	
Total	44,985	100.0	20,772	100.0	24,213	100.0	
**Educational attainment, years**							<0.01
<6	931	2.1	284	1.4	647	2.7	
6–9	20,450	45.5	8,847	42.6	11,603	47.9	
10–12	14,932	33.2	6,634	31.9	8,298	34.3	
≥13	7,717	17.2	4,676	22.5	3,041	12.6	
Missing data	955	2.1	331	1.6	624	2.6	
Total	44,985	100.0	20,772	100.0	24,213	100.0	
**Equivalent income ** **(time-invariant variable), Japanese yen**							<0.01
<2 million	18,185	40.4	8,494	40.9	9,691	40	
2–4 million	14,686	32.6	7,697	37.1	6,989	28.9	
≥4 million	4,304	9.6	2,170	10.4	2,134	8.8	
Missing data	7,810	17.4	2,411	11.6	5,399	22.3	
Total	44,985	100.0	20,772	100.0	24,213	100.0	
**Marital status**							<0.01
Married	32,179	71.5	17,911	86.2	14,268	58.9	
Single	11,872	26.4	2,493	12	9,379	38.7	
Missing data	934	2.1	368	1.8	566	2.3	
Total	44,985	100.0	20,772	100.0	24,213	100.0	
**Employment status**							<0.01
Never worked	5,137	11.4	821	4.0	4,316	17.8	
Stopped working	24,639	54.8	12,692	61.1	11,947	49.3	
Currently working	9,713	21.6	5,886	28.3	3,827	15.8	
Missing data	5,496	12.2	1,373	6.6	4,123	17	
Total	44,985	100.0	20,772	100.0	24,213	100.0	
**Hypertension**							<0.01
No	16,322	36.3	7,602	36.6	8,720	36	
Yes	17,899	39.8	7,956	38.3	9,943	41.1	
Missing data	10,764	23.9	5,214	25.1	5,550	22.9	
Total	44,985	100.0	20,772	100.0	24,213	100.0	
**Diabetes**							<0.01
No	28,684	63.8	12,447	59.9	16,237	67.1	
Yes	5,537	12.3	3,111	15	2,426	10	
Missing data	10,764	23.9	5,214	25.1	5,550	22.9	
Total	44,985	100.0	20,772	100.0	24,213	100.0	
**Obesity**							<0.01
No	32,620	72.5	14,928	71.9	17,692	73.1	
Yes	1,601	3.6	630	3	971	4	
Missing data	10,764	23.9	5,214	25.1	5,550	22.9	
Total	44,985	100.0	20,772	100.0	24,213	100.0	
**Hearing impairment**							<0.01
No	31,053	69.0	14,023	67.5	17,030	70.3	
Yes	3,168	7.0	1,535	7.4	1,633	6.7	
Missing data	10,764	23.9	5,214	25.1	5,550	22.9	
Total	44,985	100.0	20,772	100.0	24,213	100.0	
**Visual impairment**							<0.01
No	28,136	62.5	13,108	63.1	15,028	62.1	
Yes	6,085	13.5	2,450	11.8	3,635	15	
Missing data	10,764	23.9	5,214	25.1	5,550	22.9	
Total	44,985	100.0	20,772	100.0	24,213	100.0	
**Drinking habit**							<0.01
Never drank	27,423	61.0	7,579	36.5	19,844	82	
Stopped drinking	1,490	3.3	1,258	6.1	232	1	
Current drinker	15,328	34.1	11,741	56.5	3,587	14.8	
Missing data	744	1.7	194	0.9	550	2.3	
Total	44,985	100.0	20,772	100.0	24,213	100.0	
**Smoking habit**							<0.01
Never smoked	25,377	56.4	5,203	25	20,174	83.3	
Stopped smoking	12,253	27.2	11,062	53.3	1,191	4.9	
Current smoker	4,779	10.6	4,020	19.4	759	3.1	
Missing data	2,576	5.7	487	2.3	2,089	8.6	
Total	44,985	100.0	20,772	100.0	24,213	100.0	
**Walking time, minutes**							<0.01
<30	9,176	20.4	1,161	5.6	8,015	33.1	
30–59	14,927	33.2	6,767	32.6	8,160	33.7	
≥60	19,705	43.8	12,679	61	7,026	29	
Missing data	1,177	2.6	165	0.8	1,012	4.2	
Total	44,985	100.0	20,772	100.0	24,213	100.0	
**Frequency of going out**							<0.01
Rarely	2,901	6.4	1,161	5.6	1,740	7.2	
About once a week	16,915	37.6	6,767	32.6	10,148	41.9	
Almost daily	24,590	54.7	12,679	61	11,911	49.2	
Missing data	579	1.3	165	0.8	414	1.7	
Total	44,985	100.0	20,772	100.0	24,213	100.0	
**Depression symptoms (GDS-15 points)**							<0.01
≤4	27,867	61.9	13,346	64.2	14,521	60	
5–9	7,831	17.4	3,802	18.3	4,029	16.6	
≥10	2,468	5.5	1,205	5.8	1,263	5.2	
Missing data	6,819	15.2	2,419	11.6	4,400	18.2	
Total	44,985	100.0	20,772	100.0	24,213	100.0	
**Receiving emotional support**							<0.01
No	2,393	5.3	1,594	7.7	799	3.3	
Yes	41,212	91.6	18,580	89.4	22,632	93.5	
Missing data	1,380	3.1	598	2.9	782	3.2	
Total	44,985	100.0	20,772	100.0	24,213	100.0	
**Providing emotional support**							<0.01
No	2,855	6.3	1,637	7.9	1,218	5	
Yes	40,387	89.8	18,468	88.9	21,919	90.5	
Missing data	1,743	3.9	667	3.2	1,076	4.4	
Total	44,985	100.0	20,772	100.0	24,213	100.0	
**Receiving instrumental support**							<0.01
No	1,985	4.4	859	4.1	1,126	4.7	
Yes	41,838	93.0	19,506	93.9	22,332	92.2	
Missing data	1,162	2.6	407	2	755	3.1	
Total	44,985	100.0	20,772	100.0	24,213	100.0	
**Providing instrumental support**							<0.01
No	4,759	10.6	2,062	9.9	2,697	11.1	
Yes	37,932	84.3	17,948	86.4	19,984	82.5	
Missing data	2,294	5.1	762	3.7	1,532	6.3	
Total	44,985	100.0	20,772	100.0	24,213	100.0	
**Frequency of meeting friends**							<0.01
Never	10,146	22.6	6,194	29.8	3,952	16.3	
Once or twice a month	8,739	19.4	4,209	20.3	4,530	18.7	
About once a week	7,593	16.9	3,262	15.7	4,331	17.9	
2–3 times a week	10,350	23.0	3,842	18.5	6,508	26.9	
Almost daily	6,371	14.2	2,639	12.7	3,732	15.4	
Missing data	1,786	4.0	626	3	1,160	4.8	
Total	44,985	100.0	20,772	100.0	24,213	100.0	

GDS, Geriatric Depression Scale.

^*^Total may not become 100.0% due to rounding off.

During 242,934 person-years of follow-up (mean 1,971 and maximum 2,328 days), cognitive impairment developed in 4,198 cases (9.3%) (eTable 1). The overall incidence rate was 17.28 per 1,000 person-years. For males, during 110,947 person-years of follow up (mean, 1,950 days), cognitive impairment developed in 1,821 cases (1.6%). The overall incidence rate was 16.4 per 1,000 person-years. For females, during 131,988 person-years of follow up (mean, 1,990 days), cognitive impairment developed in 2,377 cases (1.8%). The overall incidence rate was 18.0 per 1,000 person-years.

### Outcome variable

Our primary outcome was cognitive impairment. Participants in our study were linked to Japan’s Long-Term Care Insurance (LTCI) registry, which includes a standardized in-home assessment of cognitive disability.^17^ Registration in the national LTCI scheme is mandatory, and each applicant requesting long-term care is assessed for eligibility to receive services (eg, home help) by a team of trained investigators dispatched from the certification committee in each municipality. During the home visit, each individual is assessed with regard to their ADL and instrumental ADL, cognitive functioning (eg, short-term memory, orientation, and communication), as well as mental and behavioral disorders (eg, delusions of persecution and confabulation) using a standardized protocol. Following the assessment, the applicants are classified into one of 8 levels (0: Independent to 7: Needs constant treatment in a specialized medical facility) according to the severity of their cognitive disability status. The resulting index of cognitive disability is strongly correlated with the Mini-Mental State Examination (Spearman’s rank correlation *r* = −0.73, *P* < 0.001)^18^ and level 1 of the cognitive decline scale has been demonstrated to correspond with a 0.5 point rating on the Clinical Dementia Rating scale (specificity and sensitivity 0.88, respectively).^19^ The initial certification is valid for 6 months, after which periodic re-assessments are conducted every 12 months.

In the present study, we defined our outcome as being certified as level 2 or higher (a state in which a subject at least manifests some symptoms, behaviors, or communication difficulties that might hinder daily activities).^20^^,^^21^

### Explanatory variable

First, participants were asked “Do you have any hobbies, or are you taking any lessons?” If they answered “yes,” they were asked to choose all activities they were engaged in from among the 25 choices mentioned on the questionnaire. Among all the activities included in the questionnaire, we defined cultural engagement as activities that did not involve physical activities (eg, golf, ground golf, gate ball, and walking/jogging), nor playing games, such as mahjong or interacting with the PC. Thus, among intellectual-cultural or cognitive leisure activities described in previous studies,^5^^–^^12^^,^^22^ reading books, magazines, and/or newspapers; crafts; and painting were selected as representing forms of cultural engagement. Traditional Japanese cultural activities, including poetry composition (*haiku*), calligraphy, and tea ceremony/flower arrangement, also met our criteria.

As for reading, we divided respondents into three groups based on the results of the survey on their hobbies and their answers to two questions: “Do you read newspapers?” and “Do you read books or magazines?” The three groups were as follows: 1) those who read books, magazines, and/or newspapers and stated that reading was a hobby; 2) those who read books, magazines, and/or newspapers but stated that reading was not a hobby; and 3) those who did not read (control group).

### Covariates and mediators

Following previous reports, we included basic demographic information, including age (65–69, 70–74, 75–79, 80–84, or ≥85 years),^19^ educational level (<6 years, 6–9 years, 10–12 years, or ≥13 years),^1^ household equivalized income (low: <2,000,000 yen, middle: 2,000,000–3,999,999 yen, or high: ≥4,000,000 yen),^23^ marital status (married, widowed/divorced, or unmarried), and employment status (employed, not working [never been employed or retired]).^24^ We also included other potentially modifiable risk factors for dementia that could influence participation in cultural activities (ie, variables that could confound the association between our exposure and outcome), such as hypertension, diabetes, obesity, hearing impairment, smoking habit, physical inactivity, and depression.^1^ We evaluated physical activity in terms of hours of walking per day and frequency of going out. Depressive symptoms were measured by the Geriatric Depression Scale-15 (GDS-15), with mild depression defined as a score of ≥5 points and severe depression as ≥10 points.^25^ We included drinking habit as an additional covariate,^26^ as well as visual impairment because it can affect reading habits.

We also assessed variables that could potentially *mediate* the association between cultural engagement and cognitive impairment. Social support is a potential mediator (ie, engagement in cultural activities increases social interaction with other people), thereby raising the probability of receiving/giving social support to others.

Following previous JAGES cohort studies, social support was assessed in terms of four variables: receiving or providing emotional support, and receiving or providing instrumental support.^20^^,^^25^^,^^27^ We assessed emotional support by asking “Do you have someone who listens to your concerns and complaints?” and “Do you listen to someone’s concerns and complaints?” We measured instrumental support by asking “Do you have someone who looks after you when you are sick and confined to the bed for a few days?” and “Do you look after someone when he/she is sick and confined to the bed for a few days?” We also included frequency of contact with friends (never, once or twice a month, about once a week, two to three times a week, or almost daily).

### Statistical analysis

We calculated descriptive statistics for all variables and confirmed gender differences through the chi-square test. A Cox proportional hazards model was employed to determine the association between cultural engagement variables and incident cognitive impairment. The interaction term between gender and cultural engagement was not statistically significant. Therefore, we performed the analyses without gender stratification. The cultural engagement variables were added separately. In model 1, we statistically adjusted for age, education, equivalent income, marital status, employment status, hypertension, diabetes, obesity, hearing impairment, visual impairment, drinking habit, smoking habit, hours of walking per day, frequency of going out, and depression. In model 2, we added social support and network as potential mediators of the association between cultural engagement and cognitive impairment.

In the analyses, we excluded respondents who developed cognitive impairment within 1 year of the baseline questionnaire to exclude reverse causality.

The significance level was set at *P* < 0.05. Statistical analyses were performed using IBM^®^ SPSS^®^ Statistics V25 (IBM Corp, Armonk, NY, USA).

### Ethical considerations

The JAGES protocol was reviewed and approved by the Ethics Committee on Research of Human Subjects at Nihon Fukushi University (approval No. 1005) and the Ethics Committee at the Chiba University Faculty of Medicine (approval No. 2493).

## RESULTS

### Gender differences in cultural engagement

The chi-square test revealed the existence of gender differences in each category (Table 1 and Table 2). Male and female respondents reported different activity profiles (Table 2). Males reported slightly higher engagement in intellectual activities (eg, reading books, magazines, and/or newspapers) compared to females, regardless of whether reading was a hobby. However, females showed higher involvement in other forms of cultural engagement, including creative activities (eg, crafts and painting) and traditional cultural activities (eg, poetry composition [*haiku*], calligraphy, and tea ceremony/flower arrangement).

**Table 2.** Types of cultural engagement

Cultural Engagement	All (*n* = 44,985)		Male (*n* = 20,772)		Female (*n* = 24,213)		*P*-value

	*n*	%	*n*	%	*n*	%	
**Intellectual Activities**							
Reading books, magazines, and/or newspapers							<0.01
Don’t read	2,407	5.4	743	3.6	1,664	6.9	
Read but it is not a hobby	35,413	78.7	16,432	79.1	18,981	78.4	
Read as a hobby	7,165	15.9	3,597	17.3	3,568	14.7	

**Creative Activities**							
Crafts							<0.01
Not a hobby	41,220	91.6	20,492	98.7	20,728	85.6	
Hobby	3,765	8.4	280	1.3	3,485	14.4	
Painting							<0.01
Not a hobby	43,159	95.9	20,175	97.1	22,984	94.9	
Hobby	1,826	4.1	597	2.9	1,229	5.1	

**Traditional Cultural Activities**							
Poetry composition (*haiku*)							<0.01
Not a hobby	43,861	97.5	20,356	98.0	23,505	97.1	
Hobby	1,124	2.5	416	2.0	708	2.9	
Calligraphy							<0.01
Not a hobby	43,312	96.3	20,267	97.6	23,045	95.2	
Hobby	1,673	3.7	505	2.4	1,168	4.8	
Tea ceremony/flower arrangement							<0.01
Not a hobby	43,788	97.3	20,697	99.6	23,091	95.4	
Hobby	1,197	2.7	75	0.4	1,122	4.6	

### Cultural engagement in relation to cognitive impairment

The results from models 1 and 2 of the Cox proportional hazards analyses are depicted in Table 3, including HRs and 95% CIs for the outcomes and covariates. Model 2 includes the 15 variables from model 1 and an additional 5 variables related to social support and network.

**Table 3.** Risk of cognitive impairment according to frequency of participation in cultural engagement at baseline (excluding respondents whose follow-up periods were ≤1 year, *n* = 44,985)

Cultural Engagement	Model 1	Model 2
	**Hazard Ratio for Cognitive Impairment** **(95% CI)**	
**Intellectual Activities**		
Reading books, magazines, and/or newspapers		
Don’t read	1.00	1.00
Read as a hobby	0.72 (0.63–0.82)	0.75 (0.66–0.85)
Read but it is not a hobby	0.71 (0.64–0.78)	0.72 (0.65–0.80)

**Creative Activities**		
Crafts		
Not a hobby	1.00	1.00
Hobby	0.69 (0.61–0.79)	0.71 (0.62–0.81)
Painting		
Not a hobby	1.00	1.00
Hobby	0.78 (0.65–0.94)	0.80 (0.66–0.96)

**Traditional Cultural Activities**		
Poetry composition (*haiku*)		
Not a hobby	1.00	1.00
Hobby	0.93 (0.78–1.12)	0.97 (0.81–1.17)
Calligraphy		
Not a hobby	1.00	1.00
Hobby	0.91 (0.77–1.08)	0.93 (0.79–1.10)
Tea ceremony/flower arrangement		
Not a hobby	1.00	1.00
Hobby	0.96 (0.78–1.18)	1.00 (0.81–1.22)

CI, confidence interval.

^*^Model 1 was adjusted for age, education, equivalent income, marital status, employment status, hypertension, diabetes, obesity, hearing impairment, visual impairment, drinking habit, smoking habit, hours of walking per day, frequency of going out, and depression; model 2 includes the variables in model 1, receiving or providing emotional support, receiving or providing instrumental support, and frequency of meeting friends.

**Figure 2.**
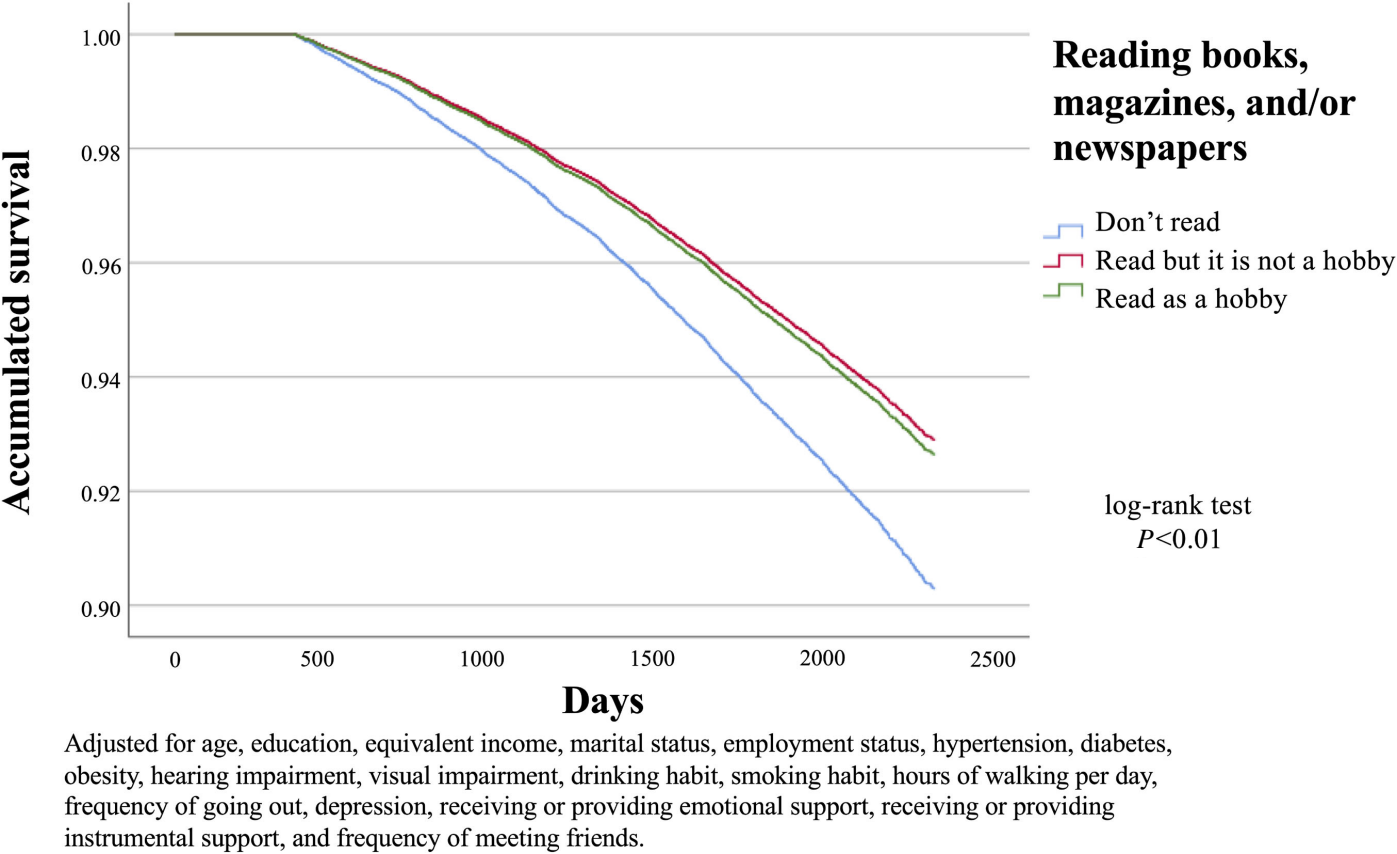
Kaplan-Meier curves for the cumulative risk of developing cognitive impairment according to whether participants read books, magazines, and/or newspapers, and whether or not reading was a hobby.

Intellectual activities (eg, reading books, magazines, and/or newspapers) were significantly related to reduced risk of cognitive impairment after adjustment for all covariates. In model 2, for those who read and stated that reading was their hobby, the HR was 0.75 (95% CI, 0.66–0.85). Those who read but did not consider reading a hobby showed a similar trend in the risk of cognitive impairment (HR 0.72; 95% CI, 0.65–0.80). Figure 2 depicts the Kaplan-Meier curves for the cumulative risk of developing cognitive impairment according to whether participants read books, magazines, and/or newspapers, and whether or not reading was a hobby.

Involvement in creative activities such as crafts (HR 0.71; 95% CI, 0.62–0.81) and painting (HR 0.80; 95% CI, 0.66–0.96) was also related to a significant decline in the risk of cognitive impairment. Writing short poems (HR 0.93; 95% CI, 0.78–1.12), calligraphy (HR 0.91; 95% CI, 0.77–1.08), and tea ceremonies/flower arrangement (HR 0.96; 95% CI, 0.78–1.18) were associated with reduced HRs of cognitive impairment in model 1; however, none reached statistical significance. Engagement in Japanese traditional cultural activities was not significantly associated with the risk of cognitive impairment.

The development of cognitive impairment according to the number of cultural engagement forms at baseline (eTable 2) and the risk of cognitive impairment according to the number of cultural engagement forms at baseline (eTable 3) are shown in the supplemental materials.

## DISCUSSION

Of the three types of cultural engagement we examined, in a large sample of Japanese older adults, intellectual activities (eg, reading books, magazines, and/or newspapers) and creative activities (eg, crafts and painting) were significantly protectively associated with cognitive impairment, while involvement in traditional cultural activities was not significantly associated with the risk of cognitive impairment.

In the present study, we showed that being engaged in reading books, magazines, and/or newspapers was associated with a lower risk of subsequent cognitive impairment. Participation in crafts or painting was also related to a decreased risk of cognitive impairment. These findings were robust even after adjustment for potential confounding variables, such as age, educational level, health status, depressive symptoms, physical exercise level, and social capital. This study, conducted using population-based data, suggests that there may be potential benefits of cultural engagement activities at the population level.

Our findings are consistent with previous reports showing the efficacy of reading in the context of cognitive impairment prevention. Although many researchers have grouped reading with intellectual-cultural or cognitive leisure activities, some studies have directly clarified the relationship between reading and dementia. Geda et al reported that reading books was associated with decreased odds of mild cognitive impairment.^11^ Verghese et al reported that those who read several times per week had a decreased risk of dementia compared to those who read once per week or less frequently.^10^

Our results suggested that respondents who were engaged in reading had a lower risk of cognitive impairment than those who do not, whether or not reading was a hobby. Reading itself may enhance brain stimulation. However, this cannot be conclusively stated, as the JAGES did not inquire about the frequency or purposes of reading.

We showed that those engaged in creative activities (eg, crafts and painting) had a significantly lower risk of cognitive impairment. Crafts such as knitting, quilting, and pottery are reportedly significantly associated with decreased risk of mild cognitive impairment^11^ or dementia.^9^ According to a recent survey on healthy aging in Korea,^28^ an eight-week program combining physical and recreational activity and art, including crafts (making cards, clay pendants, pressed flowers, mandala mobiles, and eco-bags), had positive effects on cognitive function evaluated with the Mini-Mental State Exam-Korean. In our study, arts and crafts programs were particularly favored by females, which is consistent with previous reports,^28^^,^^29^ although the Japanese word for “crafts” on our questionnaire covered knitting, sewing, beadwork, and quilting.

Painting has been classified as producing art,^22^ or an expressive activity.^30^ Crafts can be categorized as such because they require creativity. It is reported that a 12-week combined program that included painting helped patients with mild Alzheimer’s disease preserve their global cognitive function and improved their performance on attention tasks.^30^ Engagement in coloring or painting positively affected behavioral symptoms in patients with dementia in a nursing home.^29^ Our results suggest that painting and/or making hand-drawn postcards can also help prevent dementia in healthy older people.

It has been reported that a daily routine of 25 minutes of passive finger exercises incorporating several movements led to improved overall ADL in older adults with dementia.^31^ Finger activities through crafts or painting might have had a positive effect in helping prevent cognitive impairment.

To the best of our knowledge, this is the first study focusing on the effects of Japanese traditional cultural engagement on the risk of cognitive impairment. Disappointingly, no significant associations were found. Japanese care facilities for older adults often incorporate cultural pursuits for the rehabilitation and maintenance of functional and cognitive capacity.^32^ We expected engagement in *haiku* composition, calligraphy, and tea ceremony/flower arrangement to stimulate cognitive functions, as these activities involve mental discipline and training. Although we investigated a large sample of Japanese older people, our analysis revealed no significant association between engagement in Japanese traditional cultural activities and cognitive impairment. In theory, traditional cultural activities are enjoyed on special occasions, such as New Year’s, and may often require a significant investment of time, money, in terms of cost of lessons, and basic instruments. Therefore, although a considerable number of participants reported engaging in popular Japanese cultural activities, they might not practice these activities regularly. Routine or daily engagement in traditional cultural activities may delay cognitive deterioration.

Males reported slightly higher engagement in intellectual activities (eg, reading books, magazines, and/or newspapers) compared to females, regardless of whether reading was a hobby. Females showed higher involvement in creative activities (eg, crafts and painting), and traditional cultural activities (eg, poetry composition [*haiku*], calligraphy, and tea ceremony/flower arrangement). Therefore, even though the HRs for cognitive impairment are indistinguishable between male and female participants, it is suggested that males benefit more than females from intellectual activities at a population level, whereas females may benefit more than males from creative activities and traditional cultural activities. Gender differences were described in a prospective study of Swedish twins, which showed that greater participation in intellectual-cultural activities (eg, reading, listening to the radio, or watching television, social visits, and cultural activities, such as going to the theater and cinema) was associated with a lower risk of Alzheimer’s disease in females but not males.^8^

Recent studies have shown the association between greater participation in leisure activities and a decreased risk of dementia.^5^^,^^9^^,^^10^ In the present study, those engaged in at least one cultural activity showed a lower risk of cognitive impairment than those engaged in none. However, the risk of cognitive impairment was almost the same between those engaged in at least one cultural activity and those engaged in two or more (eTable 3).

Although the mechanisms that mediate between cultural activities and cognitive impairment remain unclear, acquired hippocampal neurogenesis can be cited to explain our results. Garthe et al demonstrated that mice living in a stimulus-rich, cognitively challenging environment demonstrated improved water maze learning and that they benefited to the extent relevant to adult hippocampal neurogenesis.^33^ This concept of acquired hippocampal neurogenesis can be supported by clinical or epidemiological studies involving humans.^13^^,^^22^ Cultural engagement could also help to preserve cognitive function by promoting social interactions (which have been independently shown to prevent the onset of dementia). However, comparing the results of model 1 and model 2, the HRs were almost the same; therefore, we could not find evidence of mediation by social support or frequency of meeting friends. Of course, reading is a solitary activity, and we did not expect to see mediation by social support. However, crafts and painting are often performed in the context of social participation. Nevertheless, the HRs did not change after adjusting for social support and network in model 2. Hence, we did not find evidence of mediation by social support, leaving the possibility that cultural engagement may enhance brain stimulation directly, thereby helping to preserve cognitive function.

Despite the importance of the findings, several limitations of our study must be noted. First, as we defined cognitive impairment based on functional impairment or behavioral/communication difficulties resulting from dementia symptoms, we did not classify the types of dementia, such as Alzheimer type, vascular dementia, or other treatable dementia. Second, since our questionnaire consisted of yes/no questions about hobbies and reading habits, we considered neither time commitment nor the frequency of cultural engagement. Finally, we examined one cohort, and verification in other cohorts, such as those including other racial or ethnic groups, is required.

### Conclusion

In conclusion, the present analyses demonstrate that certain types of cultural engagement could provide opportunities to prevent dementia in older adults. Intellectual activities, such as reading, were protectively associated with cognitive impairment. It is also possible that creative activities, such as crafts and painting, are related to a reduced risk of cognitive impairment.

In the future, there is a need for experimental and prospective longitudinal studies with longer durations and involving detailed assessment of the frequency and contents of cultural engagement and cognitive impairment to demonstrate the mechanisms underlying the results reported here. Increasing opportunities for community participation in these forms of cultural engagement through the establishment of clubs and circles may be effective in preventing dementia.

## APPENDIX A. SUPPLEMENTARY DATA

eTable 1. Sequential exclusion of respondents depending on follow-up periods

eTable 2. Development of cognitive impairment according to numbers of cultural engagement forms at baseline

eTable 3. Risk of cognitive impairment according to the numbers of cultural engagement forms at baseline
