# Supplementary material for: Cultural Engagement and Incidence of Cognitive Impairment: A 6-year Longitudinal Follow-up of the Japan Gerontological Evaluation Study (JAGES)
Source: J Epidemiol. 2021 Oct 5;31(10):545–53. doi: 10.2188/jea.JE20190337 (PMC8421199; doi:10.2188/jea.JE20190337)
Supplement: Supplementary file 1 [file je-31-545-s001.pdf]

eTable 1. Sequential exclusion of respondents depending on follow-up periods

Analysis	All		Male		Female	
	n	Respondents who developed cognitive impairment	n	Respondents who developed cognitive impairment	n	Respondents who developed cognitive impairment
Overall	46,058	4,697	21,340	2,030	24,718	2,667
Follow-up periods >1 year	44,985	4,198	20,772	1,821	24,213	2,377
Follow-up periods >2 years	43,528	3,501	19,996	1,503	23,532	1,998
Follow-up periods >3 years	41,934	2,690	19,166	1,173	22,768	1,517
Follow-up periods >4 years	40,287	1,787	18,321	777	21,966	1,010
Follow-up periods >5 years	38,501	794	17,373	323	21,128	471
Follow-up periods >6 years	11,030	102	4,937	34	6,093	68

*Definition of cognitive impairment: Level 2-7

eTable 2. Development of cognitive impairment according to numbers of cultural engagement forms at baseline

Analysis	Development of Cognitive Impairment					
	All (n=44,985)		Male (n=20,772)		Female (n=24,213)	
	Never	*Developed*	*Never*	*Developed*	*Never*	*Developed*
	n					
None	29,214	3,232	14,776	1,442	14,438	1,790
1 CE form	8,532	705	3,487	305	5,045	400
2 CE forms	2,369	198	569	60	1,800	138
3 CE forms	535	46	101	10	434	36
4 CE forms	120	13	18	3	102	10
5 CE forms	15	3	-	1	15	2
6 CE forms	2	1	-	-	2	1
Total	40,787	4,198	18,951	1,821	21,836	2,377

CE, cultural engagement.

eTable 3. Risk of cognitive impairment according to the numbers of cultural engagement forms at baseline

Cultural Engagement	Model 1			Model 2		
	All (n=44,985)	Male (n=20,772)	Female (n=24,213)	All (n=44,985)	Male (n=20,772)	Female (n=24,213)
	**Hazard Ratio for Cognitive Impairment (95%CI)**					
None	1.00	1.00	1.00	1.00	1.00	1.00
Low (1)	0.84 (0.78-0.91)	0.95 (0.84–1.07)	0.80 (0.72–0.90)	0.86 (0.79–0.93)	0.97 (0.86–1.09)	0.82 (0.74–0.90)
Middle to High (≥2)	0.84 (0.74-0.96)	0.98 (0.77–1.22)	0.81 (0.69–0.94)	0.88 (0.77–1.00)	1.01 (0.80–1.29)	0.84 (0.72–0.98)

CI, confidence interval.
